# A computationally constructed ceRNA interaction network based on a comparison of the SHEE and SHEEC cell lines

**DOI:** 10.1186/s11658-016-0022-0

**Published:** 2016-09-26

**Authors:** Jiachun Sun, Junqiang Yan, Xiaozhi Yuan, Ruina Yang, Tanyou Dan, Xinshuai Wang, Guoqiang Kong, Shegan Gao

**Affiliations:** 1 Department of Oncology, Cancer Institute, First Affiliated Hospital of Henan University of Science and Technology, Luoyang, People’s Republic of China; 2 Department of Neurology, Cancer Institute, First Affiliated Hospital of Henan University of Science and Technology, Luoyang, People’s Republic of China

**Keywords:** SHEE, SHEEC, miRNA, lncRNA, ceRNA

## Abstract

Long non-coding RNAs (lncRNAs) play critical and complicated roles in the regulation of various biological processes, including chromatin modification, transcription and post-transcriptional processing. Interestingly, some lncRNAs serve as miRNA “sponges” that inhibit interaction with miRNA targets in post-transcriptional regulation. We constructed a putative competing endogenous RNA (ceRNA) network by integrating lncRNA, miRNA and mRNA expression based on high-throughput RNA sequencing and microarray data to enable a comparison of the SHEE and SHEEC cell lines. Using Targetscan and miRanda bioinformatics algorithms and miRTarbase microRNA-target interactions database, we established that 51 miRNAs sharing 13,623 MREs with 2260 genes and 82 lncRNAs were involved in this ceRNA network. Through a biological function analysis, the ceRNA network appeared to be primarily involved in cell proliferation, apoptosis, the cell cycle, invasion and metastasis. Functional pathway analyses demonstrated that the ceRNA network potentially modulated multiple signaling pathways, such as the MAPK, Ras, HIF-1, Rap1, and PI3K/Akt signaling pathways. These results might provide new clues to better understand the regulation of the ceRNA network in cancer.

**Electronic supplementary material:**

The online version of this article (doi:10.1186/s11658-016-0022-0) contains supplementary material, which is available to authorized users.

## Introduction

Human esophageal cancer is the sixth leading cause of cancer-related death worldwide [1]. The most frequent subtype, esophageal squamous cell carcinoma (ESCC), has an obvious geographic variation in its distribution, with eastern Asia being a high incidence area [1, 2]. Most ESCC patients are diagnosed at an advanced stage, past the best opportunity for surgical treatment, and their overall 5-year survival rate is less than 15 %. By contrast, patients diagnosed at an early stage can have 10-year overall post-operative survival rates up to 95 % [3].

The immortalized human esophageal epithelial cell line SHEE and the malignantly transformed esophageal carcinoma cell line SHEEC were established and cultured in consecutive passes by Zeng Yi of the National Institute for Viral Disease Control and Prevention and Shen Zhongying of the Medical College of Shantou University [4, 5]. From a biological perspective, SHEE is similar to a primary cell line and retains its proliferation ability and differentiation potential. It retains the phenotype of primary epithelial cells, such as growth in a monolayer and anchorage-dependent cell aggregation without colony formation in soft agar or tumor formation after transplantation. SHEEC was established through induction of the SHEE cell line using 12-otetradeanoy-lphorbol-13-acetate (TPA). These two cell lines are the most appropriate models for ESCC tumorigenesis.

To date, considerable research has indicated that differential expression of miRNAs has been observed in various types of human cancers, such as lung cancer, gastric cancer, breast cancer, colorectal cancer and ESCC [6–9]. The expression of miRNAs has been used as a useful biomarker for tumor prognosis and clinical treatment. MicroRNAs can also regulate gene expression at the epigenetic regulation level. They are involved in most aspects of cellular processes, including development, differentiation, growth and apoptosis [10, 11].

Unlike small ncRNAs, increasing evidence indicates that lncRNAs play critical and complicated roles in the regulation of various biological processes, including chromatin modification, transcription and post-transcriptional processing [12–15]. Interestingly, additional studies have revealed that some lncRNAs can serve as miRNA “sponges” that inhibit interaction with their miRNA targets in post-transcriptional regulation. This means they can be considered competing endogenous RNA (ceRNA). Through gene expression data analysis, Sumazin et al. found >7000 transcripts acting as ceRNAs in glioblastoma tissues [16]. Cesana et al. [17] revealed the ceRNA function of linc-MD1, which liberates the differentiation factors *MEF2C* and *MAML1* from repression by decoying *miR-135* and -*133* to control muscle differentiation. Another lncRNA, *linc-RoR*, acts as an endogenous sponge to mediate *miR-145* regulation of self-renewal of human embryonic stem cells [18, 19]. Interestingly, *CHRF*, a lncRNA connected to cardiac hypertrophy-related factor, was found to act as an endogenous sponge of *miR-489*, directly binding to and downregulating *miR-489* expression levels and, in turn, regulating its target *Myd88* expression and hypertrophy [20].

The above studies identified a new regulatory mechanism in post-transcriptional regulation: ceRNA networks. The mechanism of the ceRNA network is that all types of RNA transcripts (lncRNA, pseudogenes and circular RNAs) could communicate with each other by competing for binding to shared miRNA-binding sites (MREs) [21].

In this study, we constructed a putative competing endogenous RNA (ceRNA) network by integrating lncRNA, miRNA and mRNA expression based on high-throughput RNA sequencing and microarray data to enable a comparison of the SHEE and SHEEC cell lines. Using Targetscan and miRanda bioinformatics algorithms and the miRTarbase microRNA–target interactions database, we established that 51 miRNAs sharing 13,623 MREs with 2260 genes and 82 lncRNAs were involved in this ceRNA network. Based on a biological function analysis, this ceRNA network may participate in the PI3K/Akt pathway and, consistent with previous reports, may play a modulating role in the regulation of stem-like cells in primary ESCC [22]. These results might provide new clues to better understand the regulation of the ceRNA network in cancer.

## Materials and methods

### Sample preparation

SHEE and SHEEC cells were cultured in Gibco MEM medium supplemented with 100 ml/l fetal bovine serum (containing 100 μg/ml penicillin and 100 μg/ml streptomycin) and incubated at 37 °C in a humidified atmosphere of 50 ml/l CO_2_. Cells were harvested after growth into a full monolayer and were kept at −70 °C until use.

### Total RNA isolation

Total RNA from each sample was extracted using a TRK-1001 Total RNA Purification Kit (LC Sciences) according to the manufacturer’s protocol. Total RNA was quantified on a NanoDrop ND-2000 (Thermo Scientific), and the RNA integrity was assessed using an Agilent Bioanalyzer 2100 (Agilent Technologies).

### μParaflo microRNA microarray assay

The microarray assay was performed by a service provider (LC Sciences). The assay started with a 2 to 5 μg total RNA sample that was 3′-extended with a poly(A) tail using poly(A) polymerase. An oligonucleotide tag was then ligated to the poly(A) tail for later fluorescent dye staining. Hybridization was performed overnight on a μParaflo microfluidic chip using a microcirculation pump (Atactic Technologies) [23, 24]. On the microfluidic chip, each detection probe consisted of a chemically modified nucleotide-coding segment complementary to the target microRNA (from miRBase 20.0, http://www.mirbase.org/) or other RNA (control or customer-defined sequences) and a spacer segment of polyethylene glycol to extend the coding segment away from the substrate.

The detection probes were made via in situ synthesis using PGR (photogenerated reagent) chemistry. The hybridization melting temperatures were balanced by chemical modifications of the detection probes. Hybridization used 100 l 6× SSPE buffer consisting of 0.90 M NaCl, 60 mM Na_2_HPO_4_, 6 mM EDTA (pH 6.8) and 25 % formamide at 34 °C. After RNA hybridization, tag-conjugating Cy3 dye was circulated through the microfluidic chip for dye staining. Fluorescence images were collected using a laser scanner (GenePix 4000B, Molecular Device) and digitized using Array-Pro image analysis software (Media Cybernetics).

Data were analyzed by first subtracting the background, and then normalizing the signals using a LOWESS filter (locally weighted regression) [25]. The miRNA differential expression based on the normalized signal was analyzed via selective application of Student’s *t* test. The significance threshold was *p* < 0.05 and fold-change >2.

### Construction of cDNA libraries and high-throughput sequencing

To construct the next-generation sequencing libraries, approximately 3 μg of total RNA was used to deplete ribosomal RNA according to the manufacturer’s protocol for the Human/Mouse/Rat Ribo-Zero rRNA Removal Kit (Epicentre/Illumina). Following purification, the poly(A)- or poly(A) + RNA fractions were fragmented into small pieces using divalent cations at elevated temperatures. The cleaved RNA fragments were reverse transcribed to construct a cDNA library using the dUTP method as described previously [26]. The average insert size for the paired-end libraries was 300 bp (±50 bp). RNA libraries were then sequenced on the Illumina HiSeq 2500 platform using 125-bp paired-end reads at LC Biotech in Hangzhou, China.

### lncRNA discovery

The RNAseq data were aligned to hg19 using TopHat v2.0.9 [27] with the default parameters. The mapped reads were assembled using Cufflinks v2.11 [28, 29]. All multiple assembled transcript files (GTF format) were then merged to produce a unique transcriptome set using the Cuffmerge utility provided in the Cufflinks package [30].

We filtered the assembled novel transcripts from the two pooled cell lines to obtain putative lncRNAs. First, identical and overlapping transcripts were merged to remove redundancy. Then, transcripts overlapping with known gene exons were removed. Only transcripts with a length >200 nt were retained. To identify and eliminate potential known lncRNA transcripts, we compared the merged transcriptome with lncRNA and protein-coding genes in the public authoritative database GENCODE [31].

To obtain a reliable dataset of putative lncRNAs, single exon models were filtered out. Next, we removed transcripts that were likely to be assembly artifacts or PCR run-on fragments according to their class code (annotated by Cuffmerge). Among the different classes defined by Cufflinks [32], only those annotated by “u”, “i”, “x”, “o” and “j” were retained. Extremely low gene expression is generally considered to be transcriptional noise [33]. On average, 79 % of the initial reads with a quality score >30 could be aligned to the hg19 assembly of the human genome sequence. We used CPC (http://cpc.cbi.pku.edu.cn) [34] and the coding non-coding index (CNCI) [35] to assess the protein-coding potential of each novel transcript. Those putative transcripts with a CPC score < −1 and a CNCI score < −1 were retained as candidate lncRNAs for further analysis.

### Transcript differential expression analysis

Expression levels of all of the transcripts, including putative lncRNAs and mRNAs, were quantified as fragments per kilobase of exon per million fragments mapped (FPKM) using the Cuffdiff program from the Cufflinks package. Differential gene expression was determined using Cuffdiff with a *p*-value of <0.05 and *q*-value of <0.05.

### Construction of ceRNA network

The putative miRNA–lncRNA interactions were evaluated using the algorithms of Targetscan version 6.2 (http://www.targetscan.org/) and miRanda version 3.3a (http://www.microrna.org/microrna/home.do). The miRNA binding-site prediction in lncRNAs was based on their full-length sequence in consideration of their non-coding properties. High-confidence miRNA–lncRNA pairs had a Targetscan context+ score percentile >50 and miRanda max energy < −20. To reduce the false positives, at least two miRNA binding-sites were retained with each lncRNA. The mRNAs that were targeted by miRNAs with experimental support were from miRTarbase (http://mirtarbase.mbc.nctu.edu.tw/). The ceRNA relationships were integrated using an in-house Perl script. The information including all of the above interactions was imported into Cytoscape software version 3.3.0 (http://www.cytoscape.org) to construct a regulatory network.

## Results

### Aberrantly expressed miRNAs

**Fig. 1:**
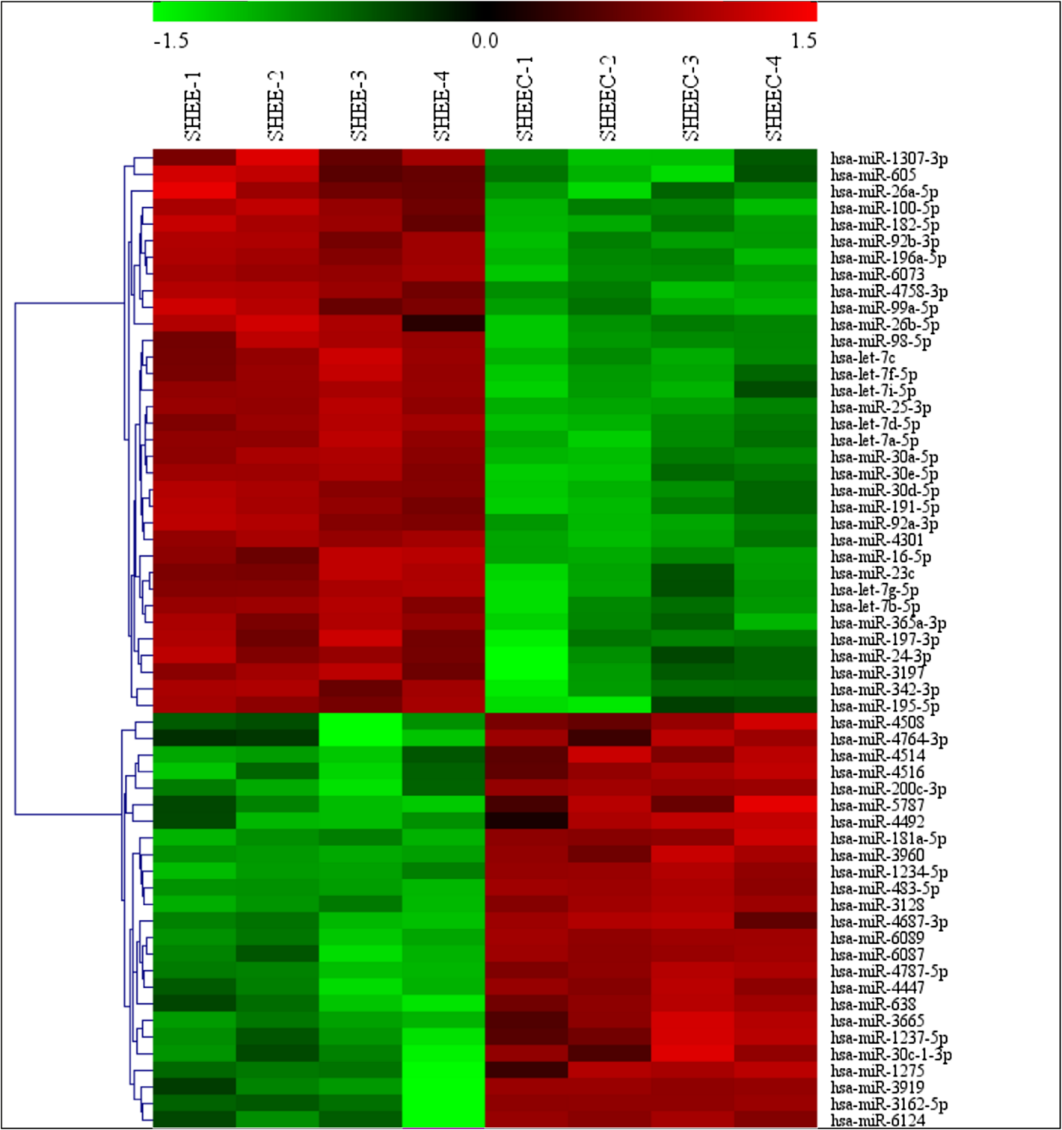
A heatmap of differentially expressed miRNAs depicting miRNAs differentially expressed in the SHEE and SHEEC cell lines with a fold-change >2.0 and *p* < 0.05

To make the preliminary determination of the miRNA expression differences between the SHEE and SHEEC cell lines, their microarray profiles were determined though microarray analysis. Of the 2019 expressed miRNAs, 59 were differentially expressed, with 25 upregulated and 34 downregulated. All differentially expressed miRNAs showed a >2.0-fold-change threshold and *p* < 0.05 (Fig. 1; Additional file 1).

### Aberrantly expressed lncRNAs and protein-coding genes

To systematically characterize the transcriptional changes between the SHEE and SHEEC cell lines, we performed high-throughput RNA sequencing according to previously published methods [36–38]. Briefly, we generated >17 million reads from strand-specific, paired-end, 125-bp RNAseq reads of total RNA depleted of rRNA from both cell populations. Using TopHat [27], an average of 95 % clean reads were mapped to the human GRCh38 genome (http://www.gencodegenes.org/releases/21.html). Using the lncRNA discovery pipeline described in the Methods section above, we detected 11,557 unique protein-coding genes and 15,932 unique lncRNAs that met the criteria of FPKM > 1 and length >200 nucleotides.

The expression levels of all transcripts were then quantified as fragments per kilobase of exon per million fragments mapped (FPKM) using the Cuffdiff program from the Cufflinks package [30]. To examine the aberrantly expressed protein-coding genes and lncRNAs, we performed differential analysis using the Cuffdiff program. By setting stringent criteria (FPKM > 3, fold-change >2, and *p* < 0.05), 5593 protein-coding genes and 6294 lncRNAs were found to be aberrantly expressed within the group comparison (Additional files 2, 3 and 4). Interestingly, the majority of the aberrantly expressed novel lncRNAs are downregulated in our dataset. The potential mechanism of dysregulation remains to be further explored.

### miRNA-binding site prediction

To establish the lncRNA–miRNA–mRNA (ceRNA) network, Targetscan 6.2 and miRanda 3.3a were used for lncRNA targeted search. Using a high confidence score (see the Materials and Methods section), the predicted MREs showed that 54 miRNAs may interact with 83 lncRNAs (Additional file 5). Based on those 54 miRNAs, we used the experimentally validated microRNA–target interactions database (miRTarbase) to search for the miRNAs’ mRNA targets. The results showed that 51 miRNAs can interact with 2260 targets (Additional file 6). Most of these targets are cancer-associated genes such as *PTEN*, *STAT3*, *VEGFA*, *KRAS*, *TP53*, *CCND1*, *CDK6*, *E2F1*, *FGFR1* and *EGFR*, with roles in cell proliferation, apoptosis, cell cycle, invasion and metastasis.

### lncRNA–miRNA–mRNA ceRNA network construction

**Fig. 2:**
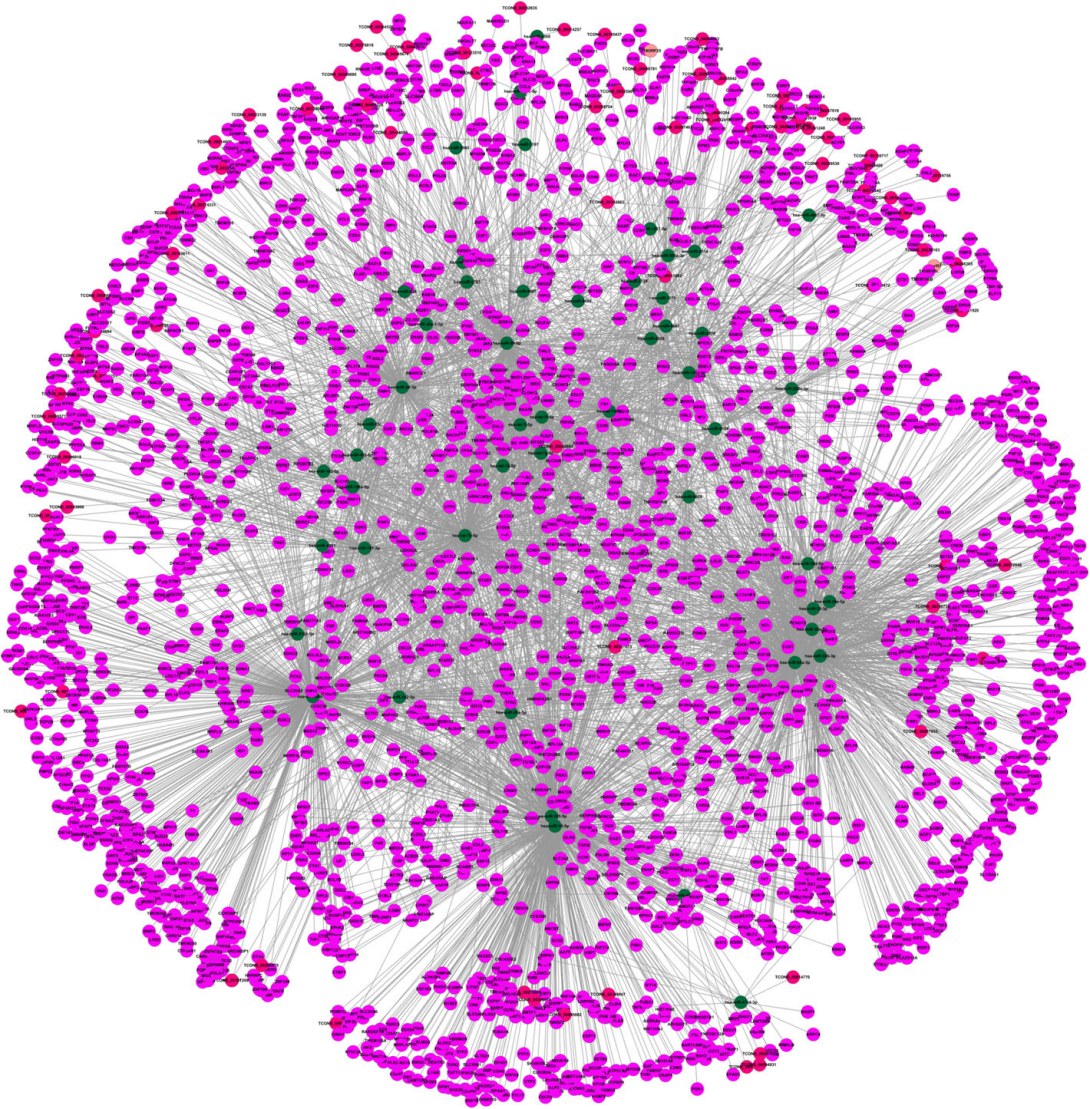
The ceRNA network. *Cadmium green* nodes represent miRNAs; *fuchsia* nodes represent genes; *bright pink* nodes represent lncRNAs

Based on the above data, the miRNA–mRNA and miRNA–lncRNA pairs were used to construct the lncRNA–miRNA–mRNA ceRNA network using an in-house Perl script. As shown in Fig. 2, 51 miRNAs shared 1,3623 MREs with 2260 unique genes and 82 unique lncRNAs in the ceRNA network (Additional file 7).

The ceRNA network was visualized by importing the above interactions into the Cytoscape software to assemble the regulation network. The genes within the above networks were further processed by gene function (GO; http://geneontology.org/) and KEGG (http://www.genome.jp/kegg/) pathway analysis.

### Gene function and pathway analysis

**Fig. 3:**
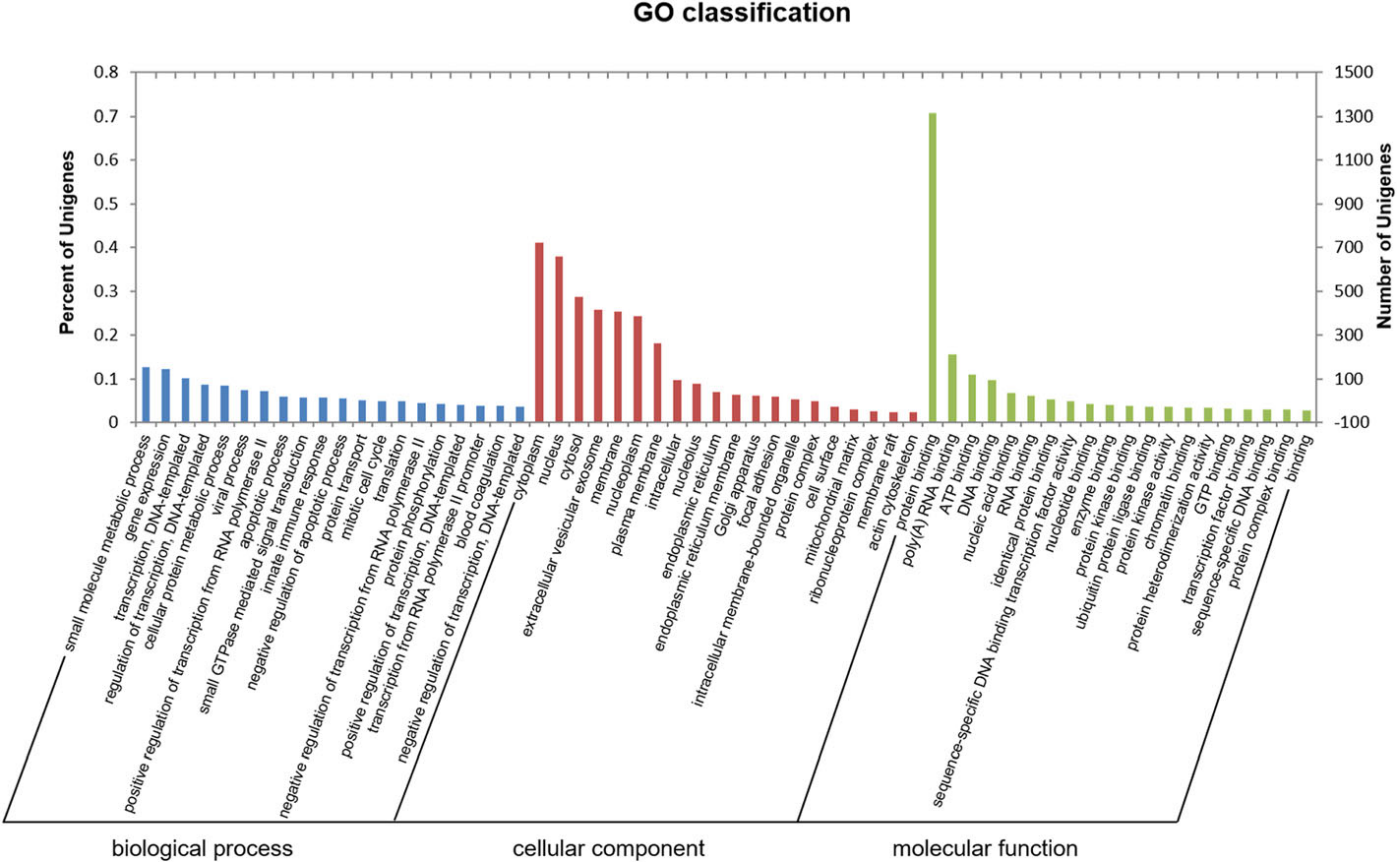
The GO enrichment analysis of the ceRNA network

To explore the function of our ceRNA network, the interrelated genes within the network were further imported to the GO consortium and KEGG pathways for annotation analysis. Based on BiNGO enrichment analysis [39], 325 GO functions were found to be the significant component relating the genes in the network, involved in processes such as small molecule metabolism, apoptosis, small GTPase-mediated signal transduction, negative regulation of apoptosis, the mitotic cell cycle, protein binding, ATP binding, DNA binding, protein kinase binding and activity, and GTP binding (Fig. 3; Additional file 8). Functional pathways analysis demonstrated that the ceRNA network potentially modulated multiple signaling pathways.

**Fig. 4:**
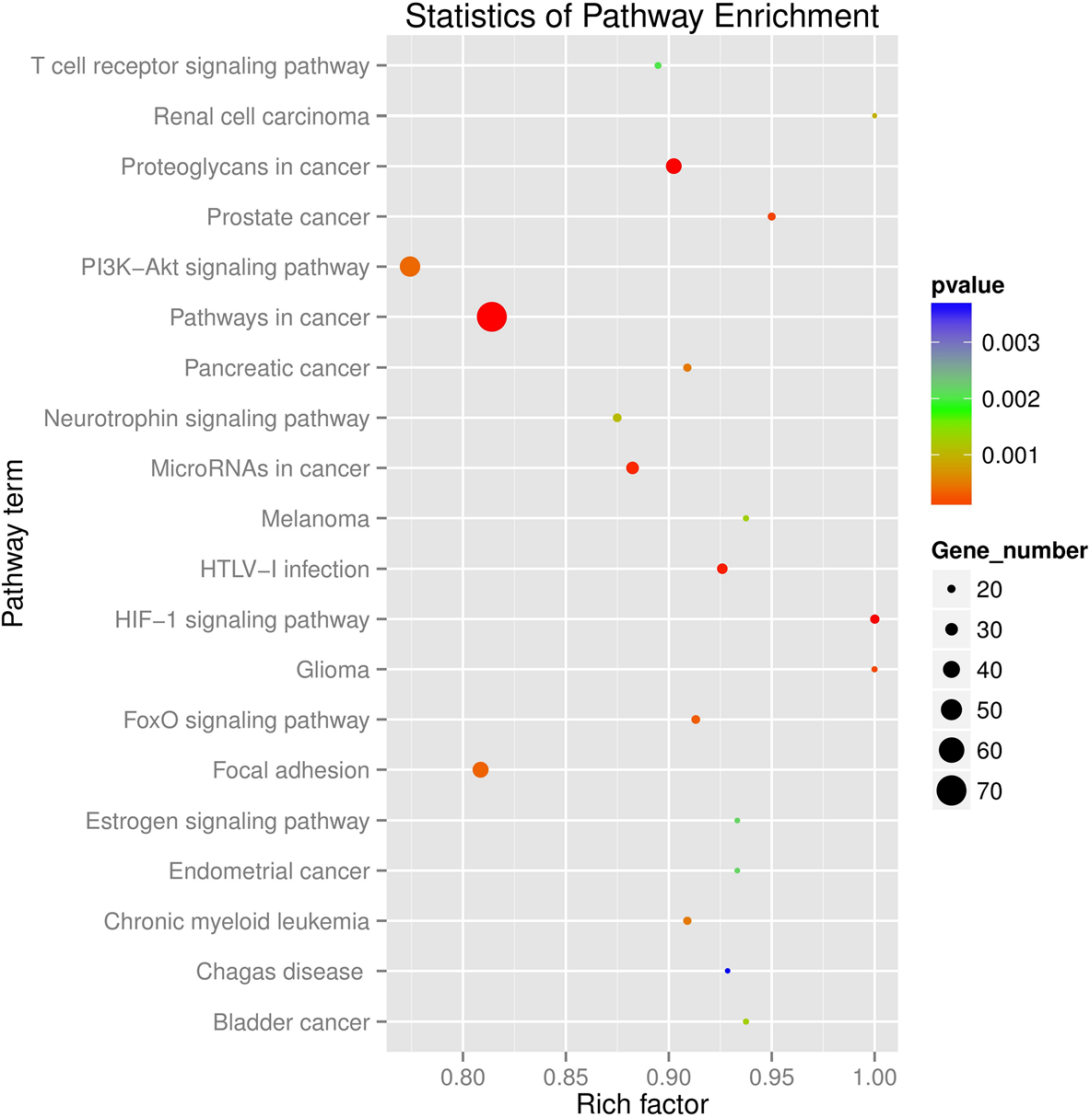
The significantly enriched KEGG terms of the ceRNA network

**Fig. 5:**
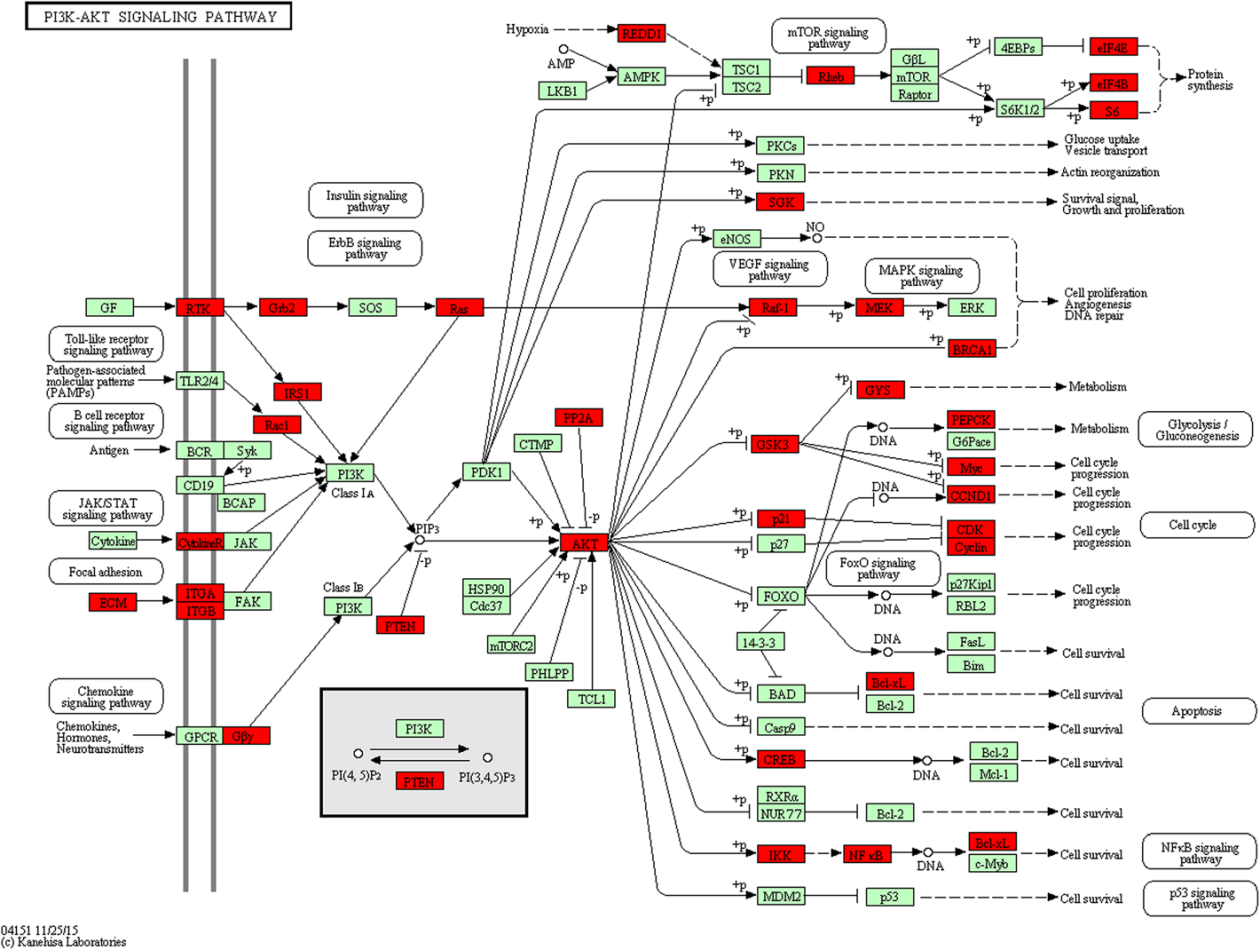
PI3K/Akt signaling pathway. The PI3K/Akt signaling pathway was the most significantly enriched biological function within the ceRNA-regulated target genes. *Red nodes* represent genes within the ceRNA network

Pathways in cancer, such as the MAPK, Ras, regulation of actin cytoskeleton, HIF-1, Rap1 and PI3K/Akt signaling pathway, were significantly associated with cell carcinogenesis (Fig. 4; Additional file 9). Consistent with the role of ceRNA regulation in the process of cell carcinogenesis [40], the PI3K/Akt signaling pathway was the biological function most significantly enriched within ceRNA-regulated genes (Fig. 5).

**Fig. 6:**
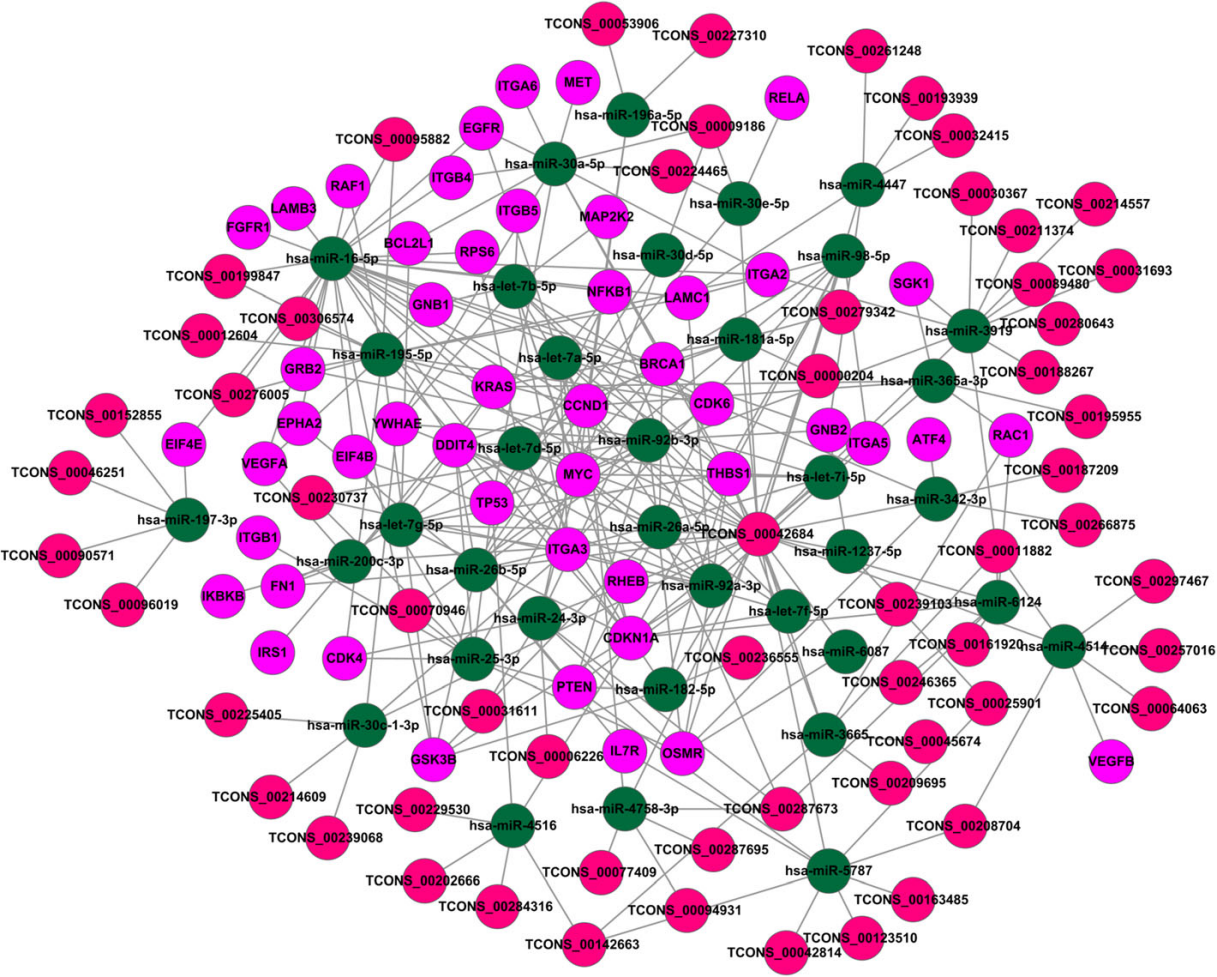
The ceRNA network modulates the PI3K/Akt signaling pathway. *Cadmium green* nodes represent miRNAs; *fuchsia* nodes represent genes; *bright pink* nodes represent lncRNAs

To further identify the relationship between these pathways and our ceRNA network, a diagram of the ceRNA modulation of the PI3K/Akt signaling pathway was created to provide useful clues regarding the mechanism associated with cell carcinogenesis. Within the pathway, 48 unique genes were enriched. Joint analysis with lncRNAs showed that 36 miRNAs share 1524 MREs with 48 unique genes and 59 unique lncRNAs (Fig. 6). The genes, including *PTEN*, *BRCA1*, *IL7R*, *EGFR*, *MET*, *ITGB4*, *ITGA2*, *ITGA6*, *EPHA2*, *VEGFA*, *GRB2*, *KRAS*, *CCDN1* and *TP53*, encode essential signaling molecules for the PTEN/PI3K/Akt pathway.

## Discussion

Accumulated studies have revealed that ceRNAs could serve as post-transcriptional regulators of protein-coding gene expression by decoying miRNAs from other target transcripts, such as lncRNAs, mRNA, pseudogenes and circular RNAs (circRNAs) [41–43]. Some studies have confirmed that ceRNAs play an important role in the regulation of gene expression in cancers such as head and neck squamous cell carcinoma, prostate cancer, papillary thyroid carcinoma, pituitary gonadotrope tumors, ovarian cancer, and chronic lymphocytic leukemia [44–46].

In this study, based on the high-throughput RNA sequencing and microarray data, we constructed a putative ceRNA network by integrating lncRNA, miRNA and mRNA expression. In our ceRNA network, lncRNA UCA1 was reported to be an independent prognostic factor associated with tumor differentiation and location [47].

Subsequent studies reported that UCA1 could upregulate 3 target genes of miR-204-5p (BCL2, RAB22A and CREB1) though competitive sponging of miR-204-5p, and then promote proliferation and chemoresistance in CRC cells. [48]. Yang et al. reported that the overexpressed UCA1 was correlated with metastasis of epithelial ovarian cancer (EOC) and functioned as a ceRNA to suppress the expression of matrix metallopeptidase 14 (MMP14) via competition for miR-485-5p. [49]. Chen et al. reported on nuclear enriched abundant transcript 1 (NEAT1) lncRNA as a novel prognostic indicator for patients with ESCC, finding that it contributes to the malignant character of ESCC [50]. NEAT1 was also reported to be a well sponge platform for many kinds of miRNA, such as miR-548 in the regulation of breast cancer cell apoptosis [51], miR-204 in regulation of epithelial-to-mesenchymal transition (EMT) and the radioresistance of NPC cells [52], and has-mir-98-5p in regulation of EGCG-induced CTR1 and cDDP sensitivity enhancement in NSCLC [53]. These specific features could potentially be used to classify lncRNAs and identify those that participate in ceRNA networks.

We found that biological function was significantly enriched with signaling pathways, small molecule metabolic processes, apoptotic processes, small GTPase-mediated signal transduction, negative regulation of the apoptotic process, mitotic cell cycle, protein binding, ATP binding, DNA binding, protein kinase binding, protein kinase activity and GTP binding, among other things (Fig. 3; Additional file 8). These signaling pathways were often altered, especially in the cancer cells, resulting in phenotypes of uncontrolled growth and increased capability to invade the surrounding tissue. These crucial molecules involved in signaling pathways represent attractive targets for cancer therapy [54–57]. Agents targeting epidermal growth factor receptor (*EGFR*), PI3K, and mTOR have been developed for interfering with their signaling functions.

Previous studies demonstrated that the PTEN/PI3K/Akt pathway was essential to side population (SP) cells thanks to its involvement in the regulation of *ABCG2* transporter function in primary ESCCs [22]. The PTEN/PI3K/Akt pathway plays a modulating role in regulating stem-like cells in primary ESCCs and may provide essential clues for the development of novel therapeutic strategies and efficient drugs.

Consistent with these studies, we found the PI3K/Akt signaling pathway was the most significantly enriched pathway, based on KEGG pathway analysis (Fig. 5; Additional file 9). *PTEN*, a well-researched protein in cancer, encodes a plasma-membrane lipid phosphatase that functions as a negative regulator of the PI3K/Akt signaling pathway [58–60].

There is more evidence that both non-coding and protein-coding transcripts regulate PTEN levels via PTEN ceRNAs and then antagonize downstream PI3K/Akt signaling [61–64, 16]. Our analysis suggested that lncRNAs like NEAT1 and TCONS_00287673 may upregulate PTEN though competitive sponging of miR-26a-5p and miR-182-5p. The regulatory mechanism of these ceRNAs needs to be further evaluated.

## Additional files

Additional file 1: Differentially expressed miRNAs. (XLS 24 kb)

Additional file 2: Differentially expressed mRNAs. (XLS 1631 kb)

Additional file 3: Differentially expressed known lncRNAs. (XLS 22 kb)

Additional file 4: Differentially expressed novel lncRNAs. (XLS 1429 kb)

Additional file 5: Relationship between miRNAs and lncRNAs. (XLS 312 kb)

Additional file 6: Relationship between miRNAs and experiment validated genes. (XLS 1416 kb)

Additional file 7: Relationship between miRNAs, experiment validated genes and lncRNAs. (XLS 6019 kb)

Additional file 8: GO enrichment analysis results of ceRNAs. (XLS 78 kb)

Additional file 9: KEGG pathway enrichment analysis results of ceRNAs. (XLS 27 kb)

## Abbreviations

ceRNA: Competing endogenous RNAs
circRNAs: Circular RNAs
CNCI: Coding non-coding index
CPC: Coding potential calculator
ESCC: Esophageal squamous cell carcinoma
FPKM: Fragments per kilobase of exon per million fragments mapped
lncRNAs: Long non-coding RNAs
MREs: miRNA-binding sites
